# The influence of spinal-pelvic parameters on the prevalence of endplate Modic changes in degenerative thoracolumbar/lumbar kyphosis patients

**DOI:** 10.1371/journal.pone.0197470

**Published:** 2018-05-15

**Authors:** Weiwei Xia, Chenjun Liu, Shuo Duan, Shuai Xu, Kaifeng Wang, Zhenqi Zhu, Haiying Liu

**Affiliations:** Department of Spinal Surgery, Peking University People’s Hospital, Beijing, China; George Washington University, UNITED STATES

## Abstract

**Background:**

The typical degeneration of the vertebral endplate shown in MRI imaging is Modic change. The aim of this study was to observe the distribution of the Modic changes of vertebral endplate in degenerative thoracolumbar/lumbar kyphosis (DTK/LK) patients and analyse the correlation between spinal-pelvic parameters and Modic changes.

**Methods:**

The imaging data of 58 patients diagnosed with DTK/LK (coronal Cobb angle<10°with sagittal imbalance) in our hospital from March 2016 to May 2017 were reviewed retrospectively. Observe the prevalence, type and distribution characteristics of Modic changes occurred at the vertebral endplate from T10 to S1;analyse the correlation between Modic changes and disc degeneration, the sagittal vertical axis (SVA), thoracic kyphosis (TK), thoracolumbar kyphosis (TLK), lumbar lordosis (LL), sacral slope (SS), pelvic tilt (PT) and pelvic incidence (PI).

**Results:**

Of the 928 intervertebral endplates from 58 patients, Modic changes occurred at 90 endplates (9.7%) of 30 patients (51.7%). 5 endplates (0.5%) of 3 patients (5.2%) were classified as type I, 68 endplates (7.3%) of 25 patients (43.1%) as type II, 17 endplates (1.8%) of 9 patients (15.5%) as type III. The location of the degenerative endplates: 2 (2.2%) superior and inferior endplates of L1, 3 (3.3%) inferior endplates of T11and T12, 4 (4.4%) superior endplates of L2, 6 (6.7%) inferior endplates of L2 and L4, 8 (8.9%) superior endplates of S1, 9 (10%) superior endplates of L3, 11 (12.2%) inferior endplates of L3 and L5 and superior endplates of L4, 12 (13.3%) superior endplates of L5. Modic changes were significantly correlated with intervertebral disc degeneration (r = 0.414, p<0.01); the amount of Modic changes were significantly correlated with LL (r = -0.562, p = 0.012), SS (r = -0.46, p = 0.048), PT (r = 0.516, p = 0.024).

**Conclusions:**

Most of the Modic changes of vertebral endplates in DTK/LK patients are type II which are prevalently located at L3/4, L4/5 and L5/S1. The Modic changes of vertebral endplates were found to be significantly correlated with disc degeneration, LL, SS, and PT.

## Introduction

One important figure of spinal degeneration shown in magnetic resonance imaging (MRI) is Modic changes. Modic changes are the signal changes of the endplates and adjacent bone marrow in MRI. Modic et al., in 1988, systematically elaborated the characteristics of Modic changes. According to the signal differences in MRI, the Modic changes includes type I, II, and III, which have different histological changes[1].

The reasons leading to Modic changes include: 1) biochemical factors, e.g., the injury of intervertebral disc and nucleus pulposus cause the release of inflammatory mediators which can lead to destruction of vertebral endplate and adjacent bone marrow; 2) the changes of spine biomechanics which easily cause the injury of the endplates. The intervertebral disc degeneration causes the structural changes of the disc which may lead to the uneven distribution of vertebral compressive loading and thus increase the likelihood of developing cracks of the endplates; the sustained compressive loading, such as obesity, can increase in the axial loading of the endplates, and excessive loading can cause micro-fractures of the endplates; the loss of normal spine curve can make the force on the intervertebral disc and endplates uneven and thus make local area exceed the normal loaded force which lead to the injury of cartilage endplate, osseous endplate and trabecular bone[2].

Spinal degenerative kyphosis is the structural deformity caused by degenerative degeneration of the spine, which is mainly manifested by the decrease or loss of the normal lordosis angle of thoracolumbar or lumbar part of the spine on the sagittal plane, and then further developing into thoracolumbar or lumbar degenerative kyphosis (TLDK/LDK) deformity[3]. The prevalence of TLDK/LDK in Asian countries is relatively high, mainly due to some special lifestyles, such as the long squat posture in agricultural work or housework[3]. The patients with degenerative kyphosis are also often associated with Modic changes of the vertebral body and severe intervertebral disc degeneration[4]. The degenerative sagittal imbalance of the spine causes the changes of axial stress of the vertebral body and the intervertebral disc resulting in the damage of the disc and endplate, and the deformed intervertebral disc and endplate may aggravate the sagittal imbalance of the spine. It is reported that the incidence of Modic changes in patients with degenerative kyphosis is higher than that in patients without kyphosis[4], but it does not indicate whether the morphological changes of the spine-pelvis are correlated with the Modic changes in the degeneration of the spine.

In this study, we focused on the influence of sagittal imbalance on the Modic changes of the vertebral body. We retrospectively analyzed the imaging data of 58 patients with degenerative thoracolumbar/lumbar kyphosis since March 2016 to May 2017, observed the Modic changes in the vertebral body, measured the sagittal spine-pelvis parameters, and analysed the results statistically. The aim was to: 1) discuss the distribution characteristics of Modic changes in patients with kyphosis (without scoliosis) in thoracolumbar and lumbar spine, and 2) analyse the correlation between the spine-pelvis sagittal imaging parameters and the Modic changes in spine degeneration.

## Materials and methods

### Study population

The medical records of 58 patients who had been diagnosed with degenerative spine kyphosis when attending our hospital from March 2016 to May 2017 were retrospectively analyzed. The patients were diagnosed according to the clinical symptoms and image assessments. The clinical symptoms include: low back pain, radicular pain, leg numbness and intermittent claudication. The image assessments include: MRI and X-ray. Imaging examination were done when the patients attended our hospital. The inclusion criteria for the study patients were: no history of tumor, tuberculosis, infection, trauma and other patients with definite pathological changes; no history of scoliosis (cobb angle of coronal scoliosis is less than 10°) and spine surgery. There were 30 males and 28 females with age ranging from 48 to 76 years enrolled in this study (mean age = 63.8 years). Eight intervertebral discs and the adjacent endplates were studied from T10 to S1. The imaging parameters of this study were determined by three independent observers (two senior spinal surgery doctors and one radiologist) who were blinded to the subject’s demographics and clinical profile. The study was approved by the Medical Ethics Committee of Peking University People’s Hospital, and all patients gave written informed consent for their information to be stored in the hospital database and used for research. This study was conducted according to the principles expressed in the Declaration of Helsinki.

The body weight were assessed by the body mass index (BMI = weight (kg)/height squared (m2)) for Asians recommended as obesity classification standard parameters by WHO in 2000[5]. The patients’ weight were assessed based on BMI: 18.5~22.9 (normal weight), 23~24.9 (overweight), and BMI≥25 (obese). The smoking habit were assessed according the method of Jensen[6]: no-smoking, mild smoking (0~19 cigarettes/day), and heavy smoking (≥20 cigarettes/day). The pain assessments include rest pain score and activity pain score. A average value of the two was calculated as the final pain score. The pain sensation was measured using a 10-cm visual analogue scale (VAS), where 0 = no pain and 10 = unbearable pain.

### Imaging examination

MRI Examination: High-resolution 1.5T MRI (Signa CVI) examination were performed in all patients when attending hospital. Sequence and parameters: sagittal T1-weighted image (repetition time (TR) 600 ms, Echo Time (TE) 13 ms), sagittal T2 weighted image (TR 2400 ms, TE 114 ms). The T1 weighted image adopts the spin echo sequence, and the T2 weighted image adopts the fast spin echo sequence. The scanning uses the surface coil, the slice width was 4mm, the interslice gap was 0.4mm, the acquisition matrix was 512 mm×256 mm.

X-ray: The standing full length of 36-inch positive lateral spine X-rays were performed in all patients with upper limb forward flexion at 60° and knee and hip joint fully stretched when attending hospital. The X-ray was used by GE healthcare System with automatic exposure control system. The voltage was 75 kV on lateral position, 85 kV on anteroposterior position and the electric current was 500 μ A.

### Imaging assessment

**Fig 1:**
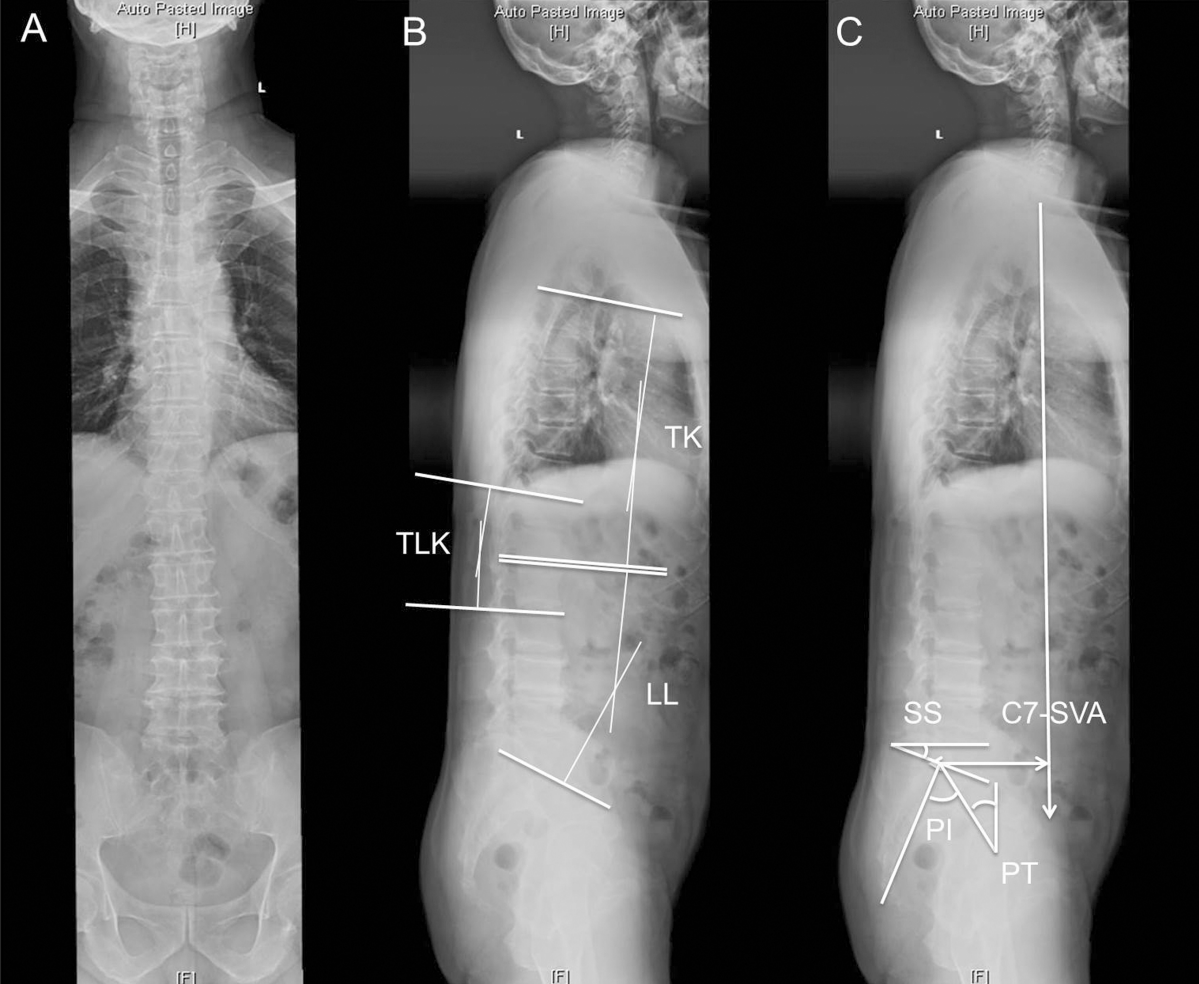
Measurements of spinal-pelvic parameters. A. Coronal plane of full-length spine X ray. There was no clear scoliosis (Cobb angle<10°). B.C. Sagittal plane of full-length spine X ray. Measurements of sagittal spinal parameters include: sagittal vertical axis (SVA), thoracic kyphosis (TK), thoracolumbar kyphosis (TLK), lumbar lordosis (LL); pelvic parameters include: sacral slope (SS), pelvic tilt (PT) and pelvic incidence (PI).

On the whole spine lateral X-rays, the measurements of the spine parameters include: 1) sagittal vertical axis (SVA), SVA was defined as the horizontal distance between the posterior corner of the sacrum and the C7 plumb line on the lateral radiograph; 2) the thoracic kyphosis (TK), TK was measured from the T4 superior end plate to T12 inferior end plate; 3) thoracolumbar kyphosis (TLK), TLK was measured from the T11 superior end plate to L1 inferior end plate; 4) lumbar lordosis (LL), LL was measured from the L1 superior end plate to S1 superior end plate. The method of measuring the angle is Cobb method. For TK, TLK, and LL, we defined lordosis was positive and kyphosis was negative (Fig 1).

On the whole spine lateral X-rays, the measurements of the pelvis parameters include:1) pelvic incidence (PI), PI was defined as the angle between the line perpendicular to the sacral plate and the line connecting the midpoint of the sacral plate to the bicoxofemoral axis; 2) sacral slope (SS), SS was the angle between the S1 superior end plate and a horizontal line; 3) pelvic tilt (PT), PT was defined as the angle between a vertical line originating at the center of the bicoxofemoral axis and a line drawn between the same point and the middle of the superior end plate of S1 (Fig 1).

**Fig 2:**
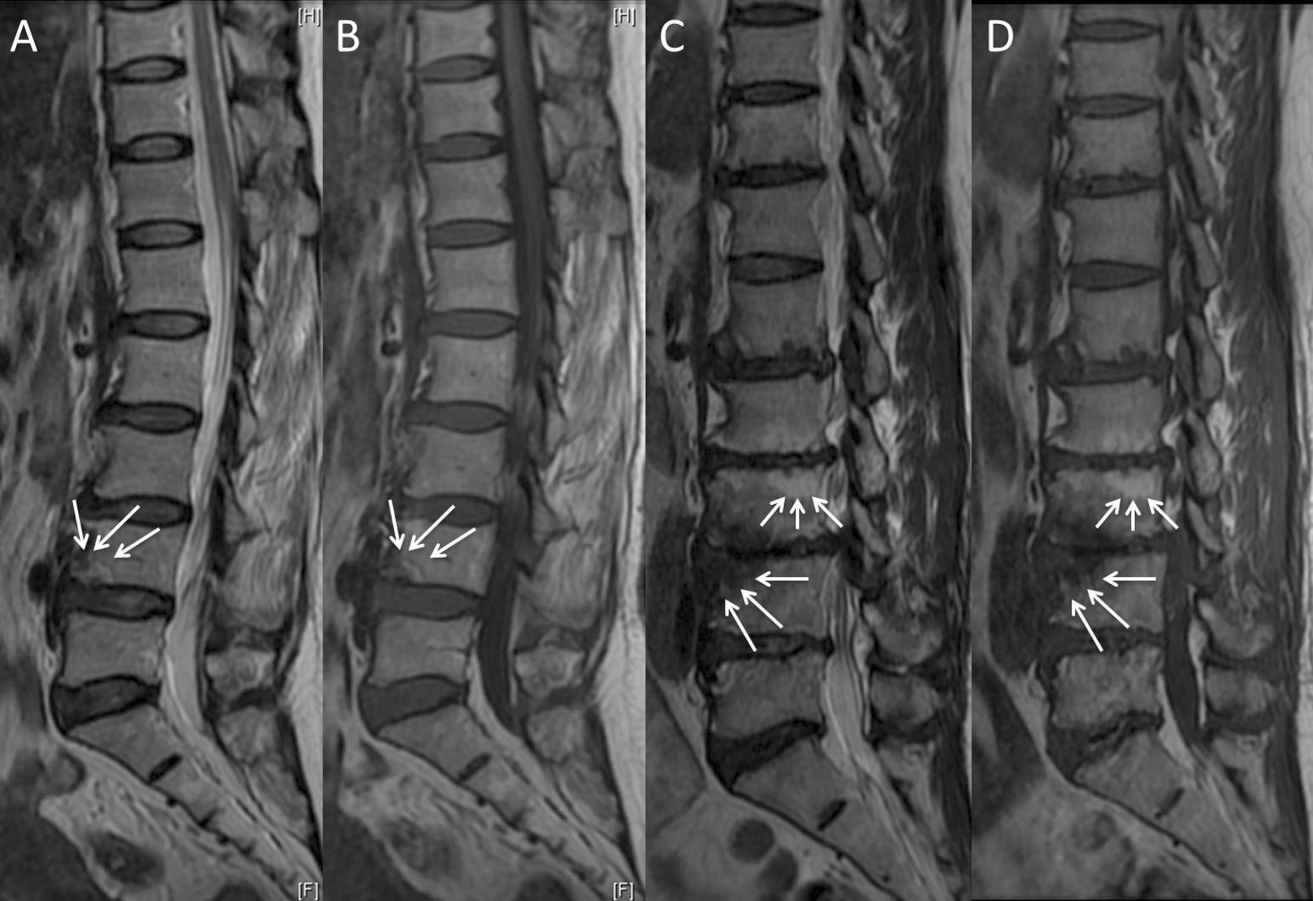
Modic changes. Modic type I change: hyperintense on T2WI (A↘), hypointense on T1WI (B↘) at inferior endplate of L4. Modic type II change: hyperintense on T2WI (C upper↘), hyperintense on T1WI (D upper↘) at superior endplate of L3. Modic type III change: hypointense on T2WI (C inferior↘), hypointense on T1WI (D inferior↘) at superior endplate of L4.

According to the classification criteria of MCs [1–3], there are five categories of endplate change. Type 0: the endplate signal is normal; Type I: the endplate shows regions that are hypointense on T1WI and hyperintense on T2WI; Type II: the endplate shows regions that are hyperintense on T1WI and isointense or hyperintense on T2WI, but the signal changes are less marked than for Type I; Type III: the endplate shows regions that are hypointense on both T1WI and T2WI (Fig 2). The degeneration of the endplates was classified into four grades based on Modic changes: type 0 is grade 1, type 1 is grade 2, type 2 is grade 3, type 3 is grade 4. The number of endplate with Modic changes from T10 to S1 were counted. The grade of degeneration of the endplates in each segment was defined as the average grade of the superior and inferior endplate in each segment.

Intervertebral disc degeneration was classified into five grades based on the T2- weighted MRI, according to the criteria of Pfirrmann[7]: Grade I: The structure of the disc is homogeneous, with a bright hyperintense white signal intensity and a normal disc height. Grade II: The structure of the disc is inhomogeneous, with a hyperintense white signal. The distinction between nucleus and anulus is clear, and the disc height is normal. Grade III: The structure of the disc is inhomogeneous, with an intermediate gray signal intensity. The distinction between nucleus and anulus is unclear, and the disc height is normal or slightly decreased. Grade IV: The structure of the disc is inhomogeneous, with an hypointense dark gray signal intensity. The distinction between nucleus and anulus is lost, and the disc height is normal or moderately decreased. Grade V: The structure of the disc is inhomogeneous, with a hypointense black signal intensity. The distinction between nucleus and anulus is lost, and the disc space is collapsed.

According to the study of Takemitsu *et al*, the patients with kyphosis were divided into the following types[8]: Type 1, degenerative flat back, which shows little lumbar lordosis and marked loss of thoracic kyphosis; Type 2, slight lumbar kyphosis combined with slight lordosis in the thoracic region; Type 3, increased lumbar kyphosis with varying degrees of thoracic lordosis; and Type 4, enlarged thoracic kyphosis which extends downward through the lower lumbar region.

### Data analysis

The correlation between the number of endplates with Modic changes and TK, TLK, LL, PI, PT and SS was analyzed by Pearson correlation test, and the relationship between the degeneration level of endplate and the degeneration level of intervertebral disc was analyzed by Pearson correlation test. The data is presented as mean values±S (standard deviation). P-value<0.05 was considered to be statistically significant. SPSS (v23.0) software was used for data analysis.

## Results

### Prevalence of Modic changes

According to Takemitsu classification method for degenerative kyphosis, 58 patients with degenerative kyphosis were enrolled in this study, which included 22 cases of type I (38%), 27 cases of type II (47%), 9 cases of type III (15%). The Modic changes occurred in 30 patients (51.7%) which included 11 cases of type I (19%), 14 cases of type II (24%), 5 cases of type III; the coronal Cobb angle of the spine was 0.8±2.4°.

The 30 patients (51.7%) with endplate Modic changes include 17 females and 13 males. In the 30 patients with Modic changes, 3 patients (10%) were in normal weight, 6 patients (20%) were overweight, 21 patients (70%) were obese. There were no or only mild smokers found in the patients with Modic changes. The VAS pain score of the patients with Modic changes was 1.0±0.2 (rest pain:0.5±0.2; activity score: 1.6±0.2; mean±SEM). 90% (27/30) of the patients had low back pain, 85% (26/30) of the patients had radicular pain, 50% (15/30) patients had leg numbness, 65% (20/30) of the patients had intermittent claudication.

**Fig 3:**
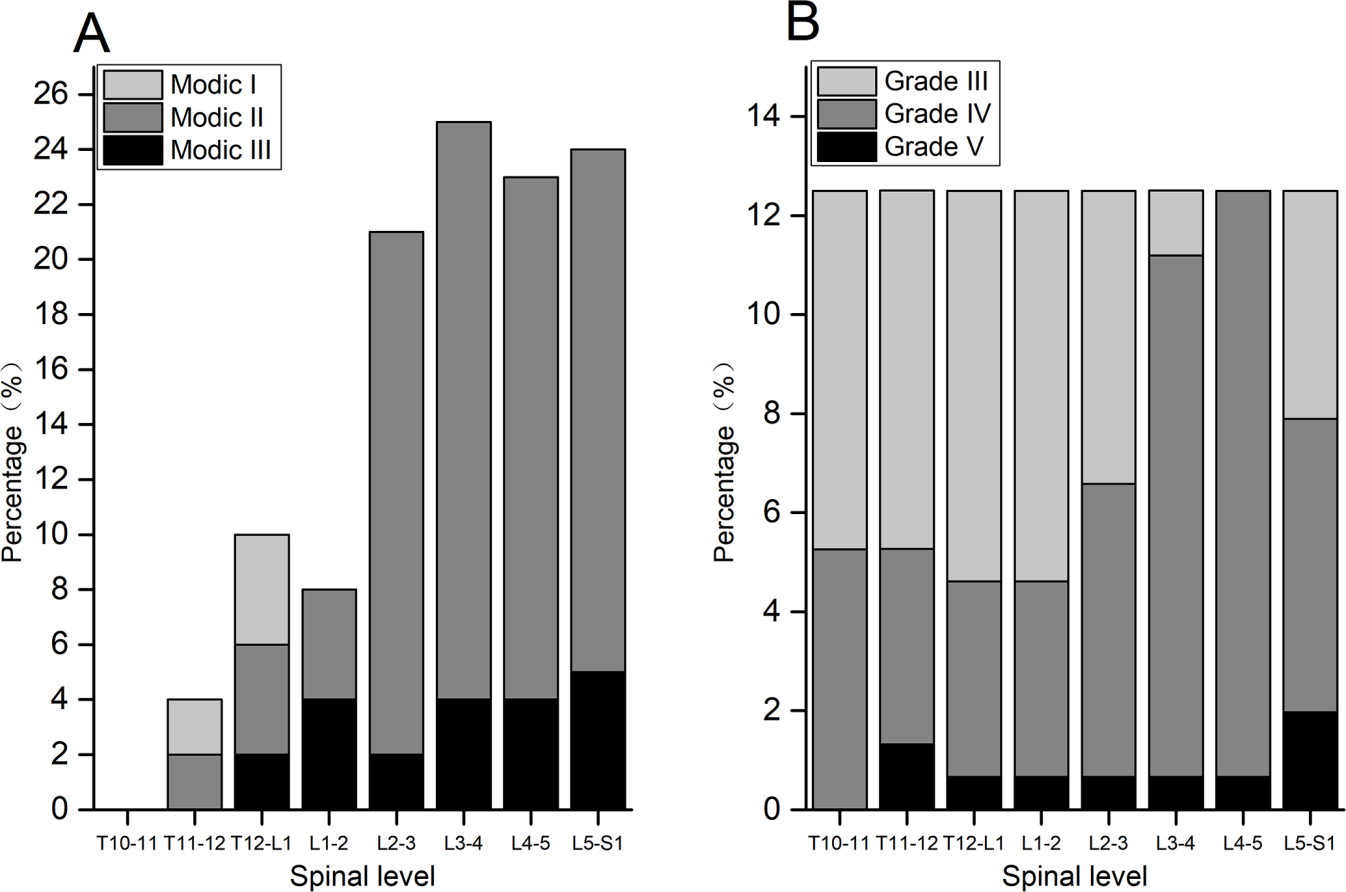
Spinal degeneration. A. The distribution of Modic changes. B. The intervertebral disc degeneration of different spinal levels.

Among the 928 vertebral endplates in 58 patients, 90 (9.7%) vertebral endplates had Modic changes. 5 (0.5%) vertebral endplates in 3 patients (5.2%) were Type I, 68 (7.3%) vertebral endplates in 17 patients (15.5%) were Type II, 17 (1.8%) vertebral endplates in 9 patients (15.5%) were Type III. The location of endplates with Modic change: 2 superior and 2 inferior endplates of L1 (2.2%), 3 inferior endplates of T11 and T12 (3.3%), 4 superior endplates of L2 (4.4%), 6 inferior endplates of L2 and L4 (6.7%), 8 superior endplates of S1 (8.9%), 9 superior endplates of L3 (10%), 11 inferior endplates of L3 and L5 (12.2%), 11 superior endplates of L4 (12.2%), 12 superior endplates of L5 (13.3%). Majority of the Modic changes occurred at the low lumbar levels (Fig 3).

Pfirrmann classification of intervertebral disc degeneration in 30 patients: in 240 intervertebral discs, there were 103 discs in grade III (43%), 129 discs in grade IV (54%), 8 in grade (3%). The disc degeneration in each spinal level: T10-T11:17 in grade III (7%), 13 in Grade IV (5%); T11-T12: 17 in grade III (7%), 10 in grade IV (4%), 3 in grade V (1%); T12-L1: 21 in grade III (9%), 9 in Grade IV (4); L1-L2: 19 in grade III (8%), 9 in grade IV (4%), 2 in grade V (0.8%); L2-L3: 14 in grade III (6%), 16 in grade IV (7%); L3-L4: 3 in grade III (1%), 27 in grade IV (11%); L4-L5: 30 in grade IV (13%); L5-S1: 11 in grade III (5%), 16 in grade IV (7%), 3 in grade V (1%) (Fig 3).

### Spine-pelvic sagittal parameters

Sagittal parameters of spine: SVA value was 79.9±12.2 mm; TK was 22.4±2°; TLK was 13.2±1°; LL was 22.2±2.4°. Pelvic sagittal parameters: SS was 23.5±2.1°; PT was 22.5±1.5°; PI was 46.1±2.0°.

### Correlation between spine degeneration and spinal-pelvic parameters

**Fig 4:**
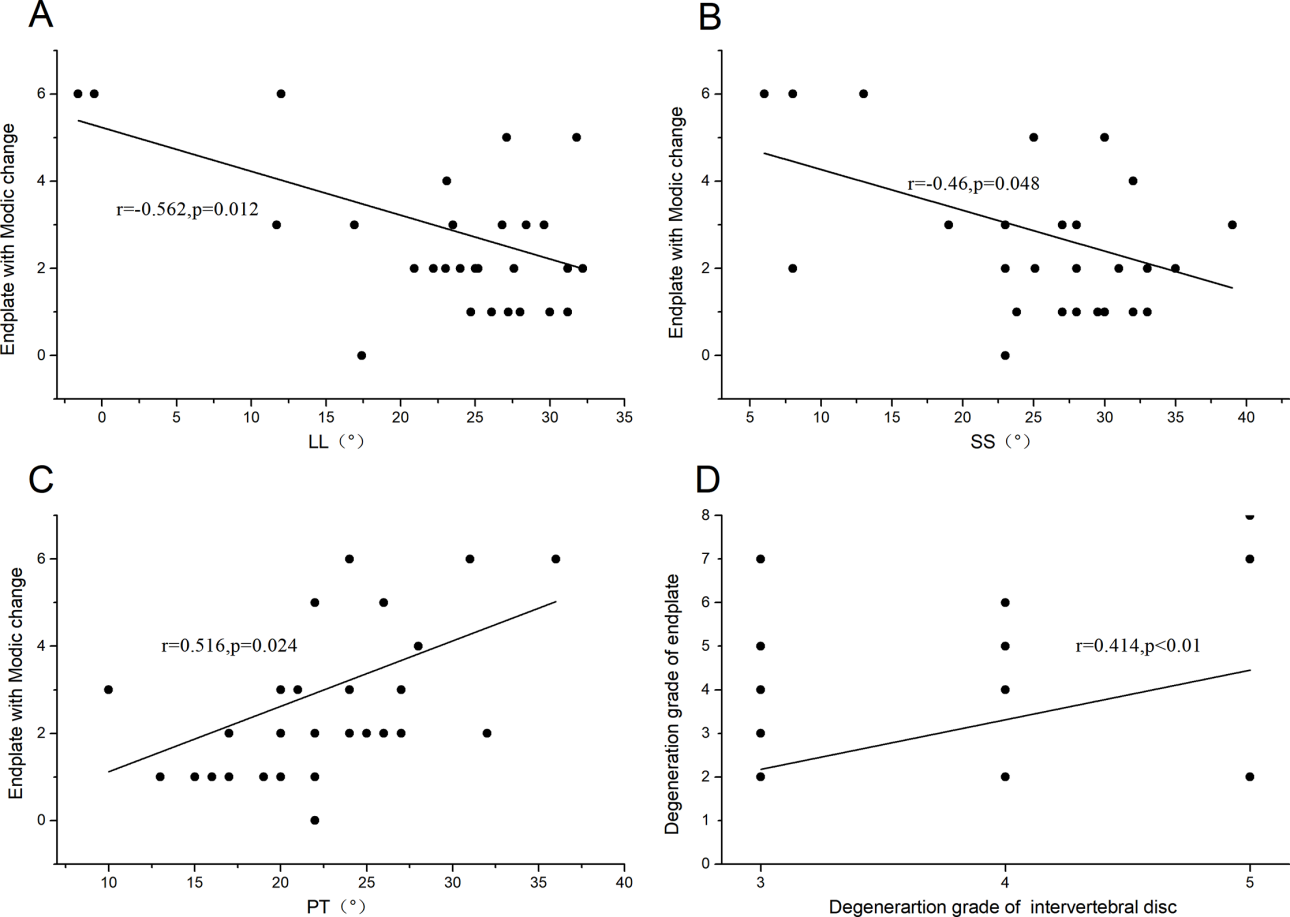
Correlation analysis. Modic changes were significantly correlated with intervertebral disc degeneration; the amount of Modic changes were significantly correlated with lumbar lordosis (LL), sacral slope (SS) and pelvic tilt (PT).

The correlation analysis between Modic changes and spinal-pelvic parameters were shown in Table 1. The grade of Modic changes was significantly correlated with the grade of intervertebral disc degeneration (r = 0.414,p<0.01). The number of endplate with Modic changes was significantly correlated with LL (r = -0.562,p = 0.012), SS (r = -0.46,p = 0.048), PT (r = 0.516,p = 0.024) (Fig 4).

**Table 1:** Correlation between Modic changes and spinal-pelvic parameters (r value, p value).

Assessments	SVA	TK	TLK	LL	SS	PT	PI	Pfirrmann
Modic changes	0.051,0.837	-0.259,0.284	-0.075,0.759	-0.562,0.012*	-0.46,0.048*	0.516,0.024*	-0.103,0.675	0.414,0.00**

SVA: sagittal vertical axis; TK: thoracic kyphosis; TLK: thoracolumbar kyphosis; LL: lumbar lordosis; SS: sacral slope; PT: pelvic tilt

*:p<0.05

**:p<0.01.

## Discussion

### Prevalence of Modic changes

The results of this study showed that the Modic changes were most common in the lower lumbar spine, and the type of change was mainly type II, which was consistent with other studies[9,10]. However, the incidence rate (51.7%) of this study was higher than that of the general population (5.8~22.4%)[9,10]. The main reason is that this study recruited mainly elderly patients, and the patients had a certain degree of degenerative deformity of the spine, so the incidence of Modic changes is significantly increased. In addition, the differences in the reported prevalence across different studies not only could be explained by the differences in subject recruitment (e.g. general population or patient population) but also due to differences in determinants of Modic changes, such as lifestyle factors (e.g. smoking, obesity, physical work, etc.) or the presence of endplate and disc pathologies[6]. Long-term heavy smoking was supposed to be a risk factor for Modic changes of the vertebral endplates in previous studies[11,12]. However, no heavy smokers were found in the kyphosis patients with Modic changes in this study. Therefore, smoking status could not affect the prevalence of Modic changes which is in agreement with Chao et al.’ findings[10]. However, the sample size of this study is relatively small which can not clarify the association between smoking and Modic changes. 90% of the kyphosis patients with Modic changes were over the normal weight indicating that obesity could increase the probability of Modic changes[10]. More female patients were found to have Modic changes compared to male patients, which may be due to the high incidence of osteoporosis in females[10]. Modic change has been shown to be highly associated with low back pain, especially Modic type I[13]. In this study, 90% of the patients with Modic changes had low back pain. However, the pain conditions in the patients with kyphosis are complex, which may be due to the compression of the dorsal root or the spinal cord with stenosis, but not only the Modic changes.

The lower lumbar spine, which is closer to the end of spine due to its anatomical position, is considered to bear a relatively high mechanical force, especially when the sagittal plane of the spine is imbalanced[12]. If the angle of deformity exceeds the normal range of spinal curve, and even the situation is decompensated, its mechanical bearing force may increase much more, accelerating the progress of degeneration. Another study reported that vascular factor (e.g., atherosclerosis) was also an independent risk factor for the Modic change of lower lumbar spine[14]. It is may be related to the diameter of the blood supply artery of L5 vertebra, which is less than that of other upper lumbar vertebrae, so the decrease of blood flow will eventually affect the repair function of the vertebral body[14].

### Relationship between Modic change and kyphosis deformity

Lumbar degenerative kyphosis (LDK) is a subtype of the flat back syndrome, mainly due to the degeneration of the spine with sagittal deformity. The sagittal deformity includes the decrease or loss of physiological lordosis of the lumbar, or even the lumbar kyphosis[15]. Some scholars have made a more explicit designation of kyphosis which should be defined as "primary degenerative sagittal imbalance (PDSI)", so LDK is only a subtype of PDSI[15]. The results of this study showed that the type 2 sagittal deformity was more common according to the Takemitsu classification method. The loss of lordosis can lead to the imbalance of the weight distribution on the spine, causing degeneration and damage to the vertebral endplate and intervertebral disc, and further aggravating the imbalance of the spine and causing pain in the patients. The patients with kyphosis may often have long-term crouching motion, so the prolonged overload force may cause the damage to the intervertebral disc and endplates. Studies have shown that the quality of life of patients with spinal deformity was significantly correlated to sagittal deformity, but not significantly correlated with the coronal deformity[16]; and the measurement of spine-pelvic parameters can help to determine the prognosis of spinal degeneration diseases[15]. In addition, it was found that, under normal standing and bearing load conditions, from the vertebra T11, the compressive force of vertebral body in the kyphosis decompensation group was significantly increased when the kyphosis angle increased by every 1° [17].

Therefore, the relationship between sagittal parameters of spinal deformity and degenerative changes of spine is particularly important. The results of this study showed that the number of endplates with Modic changes in degenerative thoracolumbar/lumbar kyphosis was negatively correlated with LL and SS, and positively correlated with PT. The correlation between Modic changes and kyphosis may be due to decreased axial decompression ability of the spine, and the increased shear force of vertebral endplate. Thus, the probability of damage to vertebral endplate and cancellous bone is increased when the spine is subjected to the external force in the perpendicular direction[4]. The degree of injury depends on the bone density of the vertebral body section; as a result, the likelihood of Modic degeneration is increased in patients with osteoporosis, especially in older women[10]. In addition, previous report showed that the Modic changes had a high incidence rate of in degenerative scoliosis, but the scoliosis was mainly structural deformity, while the sagittal kyphosis of the spine was mainly functional deformity[12]. Furthermore, the incidence of Modic changes in patients with degenerative spine kyphosis is higher than that of patients without kyphosis[4]. Therefore, the sagittal imbalance of the spine, with the absence of scoliosis, may also accelerate the damage to the vertebral endplates and adjacent bone marrow, and further aggravates the degenerative deformity.

### Relationship between Modic change and intervertebral disc degeneration

This study showed that the severity of Modic changes in vertebral endplate is positively correlated with the severity of intervertebral disc degeneration, and most of them occurred in the lower lumbar spine, which is consistent with other findings[1,18]. The causal relationship between them, however, is still uncertain in the present study. The degeneration of the vertebral endplate causes the tilt of superior and inferior edge of the vertebral body, and the intervertebral disc degeneration also causes the inconsistency of the anterior and posterior intervertebral space, which leading to the imbalance and instability of the adjacent vertebral body, thus aggravating the spinal deformity progress[19].

Studies have shown that the gene-gene interaction of interleukin-1 and metalloproteinase-3 is involved in the of type II Modic change of the vertebral endplate, and the two are also related to the degeneration of the intervertebral disc, indicating that the degeneration of intervertebral disc and endplate, to some extent, had the same genetic factor[20]. This may be a potential reason explaining the positive correlation between the two. It was reported that the damage to the endplates is prior to the disc: the calcification and destruction of the end plate reduce its penetration capacity, and thus, to a certain extent, hinder the nutritional supply of intervertebral discs, which accelerates the degeneration of intervertebral disc[21]. There was also research suggesting that Modic changes may be a secondary change in disc degeneration: the damage to the intervertebral disc or changes in the internal material can result in greater axial and torsional forces on the dynamic surface between vertebral body and intervertebral disc, leading to the formation of micro fractures of the adjacent endplates; this in turn can lead to the contact between the nucleus pulposus with the blood circulation system, causing the automatic immunity to occur, which may result in the loss of bone marrow, degeneration and hyperplasia of bone[22]. Two prospective studies for patients with sciatica reported a large increase of type I changes occurred at 24 months (discectomy) and 14 months (conservative treatments) respectively indicating that the removal of damaged disc could postpone the occurrence of Modic changes [23,24]. The disc injury was also reported to induce changes in the adjacent vertebrae, such as bone marrow depletion, degeneration and regeneration of the bone[25,26].

### Limitations

This study is a single center study and the number of cases was relatively small. This study did not record Modic changes in the cervical spine and all thoracic vertebrae. However, the types of kyphosis are diverse, the conditions are complex, and the pain was often related to many factors. Therefore, a multicenter, large data research would be more helpful to show the incidence and distribution characteristics of Modic changes in patients with only spinal kyphosis deformity.

## Conclusions

The results of this study showed that the Modic changes of degenerative thoracolumbar/lumbar kyphosis were more common in type II, and most occurred at the level of L3/4, L4/5 and L5/S1. Modic changes were positively correlated with PT and disc degeneration, and negatively correlated with LL and SS. Therefore, for the patients with sagittal imbalance of the spine, we should comprehensively analyse, and pay attention to the occurrence and distribution of Modic changes in order to better guide the treatment and prognosis, but the causal relationship of the related factors needs to be further studied.
